# Reorganization of E-cadherin into apical spot junctions mediates interlineage adhesion between epithelial and germline cells

**DOI:** 10.3389/fcell.2026.1807574

**Published:** 2026-05-11

**Authors:** Vanessa Weichselberger, Ramya Balaji, Marta Rodriguez-Franco, Anne-Kathrin Classen

**Affiliations:** 1 European Molecular Biology Laboratory, EMBL Barcelona, Barcelona, Spain; 2 Aix Marseille University, CNRS, UMR 7288, IBDM, Marseille, France; 3 Hilde-Mangold-Haus, University of Freiburg, Freiburg, Germany; 4 Faculty of Biology, University of Freiburg, Freiburg, Germany; 5 Spemann Graduate School of Biology and Medicine (SGBM), University of Freiburg, Freiburg, Germany; 6 Signalling Research Center BIOSS and CIBSS, University of Freiburg, Freiburg, Germany

**Keywords:** *Drosophila*, E-cadherin, egg chamber development, follicle cell, nurse cell

## Abstract

During development, epithelia must coordinate morphogenesis with neighboring cell lineages to drive structural remodeling of organ systems. How adhesion between epithelial and other cell types is established and maintained remains poorly understood. Using the *Drosophila* ovary as an *in vivo* model, we show that anterior follicle cells (AFCs) undergo epithelial plasticity to establish and maintain adhesion with germline nurse cells during late oogenesis. As AFCs spread over the nurse-cell compartment, adherens junctions disassemble, and E-cadherin, together with junctional partners, reorganizes into apical “spot junctions.” Formation of these junctions requires E-cadherin in both follicle and germline cells and is promoted by the expansion of the AFC apical surface. Quantitative imaging reveals that spot junctions form a uniformly spaced lattice that remains stable as the AFC-nurse cell interface enlarges. Functionally, these E-cadherin-based junctions are essential to maintain soma–germline adhesion, enabling full envelopment and clearance of nurse cell remnants by AFCs during late oogenesis. Our findings uncover a mechanism by which an epithelium repurposes its apical membrane into a specialized adhesive surface, providing a paradigm for the emergence of interlineage adhesion in developing tissues.

## 1 Introduction

Epithelia are continuous sheets of polarized cells that function as selective barriers in multicellular organisms. Classical epithelia display apical–basal polarity, with the apical domain facing an external or luminal environment and the basal surface anchored to an extracellular matrix. Cohesion of an epithelial sheet is maintained by apicolateral adherens junctions, in which E-cadherin provides homophilic adhesion and links to the cortical actin cytoskeleton, thereby integrating mechanical forces across the epithelial cell network. Epithelial tissues can undergo substantial remodeling of their architecture in response to developmental, physiological, or pathological cues. This remodeling encompasses reorganization of polarity, adhesion, and cell shapes, which underlie the plasticity of epithelia, their functional diversification, and terminal differentiation (Takeichi, 2014; Buckley and St Johnston, 2022; Mira-Osuna and Borgne, 2024).

A particularly understudied aspect of epithelial plasticity is the capacity of epithelial cells to establish functional adhesions with non-epithelial cell types, for example, neurons, immune cells, or germline cells, to create multilineage epithelial tissues *in vivo* with dynamic response states. While our understanding of inter-epithelial cell adhesion has advanced, the molecular basis and functional consequences of interactions with non-epithelial cell types remain poorly understood (Dobens and Raftery, 2000; Tanentzapf et al., 2000; Horne-Badovinac and Bilder, 2005). In classical epithelial cells, the apical surface faces a luminal space, yet in gametogenesis, the apical membrane of follicular epithelia is directly apposed to germline cells, a distinct lineage. Such configurations would require the reorganization of the apical surface into specialized signaling or adhesive structures, but the molecular mechanisms underlying this apical diversification remain poorly defined.

The *Drosophila* ovary provides a powerful *in vivo* model to investigate interactions between epithelial and non-epithelial cell types. During *Drosophila* oogenesis, as in mammalian species (Lei and Spradling, 2016; Doherty et al., 2022; Niu and Spradling, 2022; Spradling et al., 2022), an oocyte develops in a germline-derived syncytial cyst connected to nurse cells (Horne-Badovinac and Bilder, 2005; Duhart et al., 2017) and surrounded by a somatic follicular epithelium. Importantly, the basal surface of the follicular epithelium contacts an outward-facing basement membrane, while the apical surface is directly juxtaposed to the germline cells. This raises the question of how the epithelium functionalizes its apical surface to coordinate its morphogenesis with the underlying germline cells.

**FIGURE 1:**
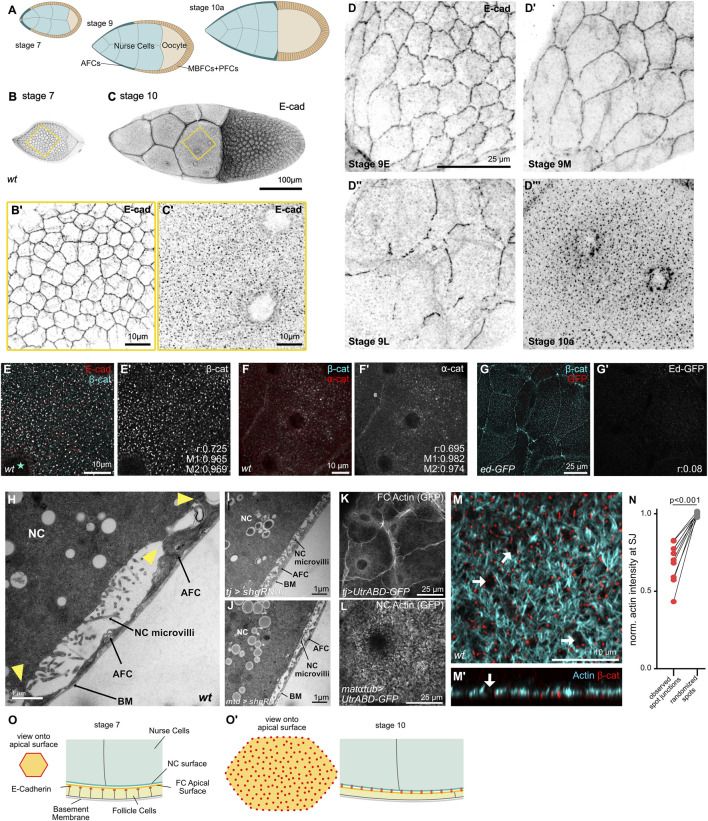
Intra-epithelial adherens junctions are remodeled into germline–soma spot junctions. **(A)** Schematics of egg chamber cross-sections from stages 7–10A. Anterior follicle cells (AFCs, dark blue) spread over nurse cells (light blue) during stage 9. **(B,C)** Maximum projections of stage 7 **(B,B’’)** and stage 10 **(C,C’)** egg chambers stained for E-cadherin. Insets **(B’,C’)** show the apical surface of follicle cells in contact with nurse cells [yellow frames in **(A,B)**]. **(D)** Maximum projections of the apical surface of AFCs at stage 9 (early, mid, and late) and stage 10A, stained for E-cadherin. Intra-epithelial AFC junctions fragment during stage 9 and E-cadherin redistributes into apical clusters by stage 10A. **(E–G)** Maximum projections of AFCs in a stage 10A egg chamber stained for E-cadherin **(E)**, β-catenin **(E–G)**, α-catenin **(F)**, or expressing *echinoid(ed)-GFP* **(G)**. Mean Pearson’s correlation coefficient and Mander’s M1 and M2 for co-localization are reported (E-cad/β-cat n = 18 cells; β-cat/α-cat n = 4 cells, and β-cat/Ed n = 7 cells). **(H)** TEM image of AFC–nurse cell interface in a *wild-type (wt)* stage 10A egg chamber. Yellow arrowheads point to cell-cell contacts between AFCs and nurse cells. **(I,J)** TEM images of AFC–nurse cell interface in a stage 10A egg chamber expressing *shg-RNAi (E-cadherin RNAi)* in follicle cells [**(I)** *TJ-GAL4*] or germline cells [**(J)** *MTD-GAL4*]. **(K,L)** Maximum projections of egg chambers expressing UAS-*utrABD-GFP* in follicle cells [**(K)** *TJ-GAL4*] or germline cells [**(L)** matαtub-GAL4]. utrABD-GFP is an actin binder composed of the actin-binding domain (ABD) of human utrophin fused to GFP (Rauzi et al., 2010). **(M)** Airyscan image of the AFC–nurse cell interface in a stage 10B egg chamber stained for F-actin and β-catenin. An orthogonal view is shown in **(M’)**. **(N)** Paired t-test of actin intensity at spot junctions: observed vs. digitally randomized distributions (see Supplementary Figure S2 and Methods). Each data point represents the mean intensity of actin at the spots for one egg chamber, *n* = 10 egg chambers, N = 2. **(O)** Schematics of the apical surface (orange) and cross-section of follicle cells (yellow) at stages 7 and 10A. E-cadherin (red) shifts from a continuous junctional belt **(O)** to apical clusters facing nurse cells **(O’)**. NC, nurse cell; AFC, anterior follicle cell; and BM, basement membrane.

During early oogenesis, the follicular epithelium differentiates into distinct subpopulations, giving rise to three major populations, namely, anterior follicle cells (AFCs), main body follicle cells (MBFCs), and posterior follicle cells (PFCs) (Dobens and Raftery, 2000; Horne-Badovinac and Bilder, 2005; Roth and Lynch, 2009). During stage 9, these epithelial subpopulations reposition over the germline to create precisely matched interlineage associations: PFCs and MBFCs reposition over the oocyte surface, while AFCs spread out to cover the entire nurse cell compartment (Figure 1A) (Dobens and Raftery, 2000; Horne-Badovinac and Bilder, 2005; Grammont, 2007; Kolahi et al., 2009; Roth and Lynch, 2009; Chlasta et al., 2017; Balaji et al., 2019; Lamire et al., 2020; Weichselberger et al., 2022). This spatial arrangement is essential for all successive developmental steps and, therefore, must be maintained into late oogenesis. During late oogenesis, nurse cells undergo nurse cell dumping, a process in which nurse cells transfer their cytoplasmic contents into the oocyte (Huelsmann et al., 2013; Timmons et al., 2016; Imran Alsous et al., 2021; Logan et al., 2022; Niu and Spradling, 2022; Spradling et al., 2022; Giedt and Tootle, 2023). While nurse cells transfer their cytoplasmic content into the oocyte, AFCs envelop the nurse cells and eventually remove their remnants via phagoptosis (Etchegaray et al., 2012; Peterson and McCall, 2013; Timmons et al., 2016; Lebo and McCall, 2021). Thus, the mechanical and signaling interactions of AFCs and nurse cells are essential for successful oogenesis.

Although adhesion between germline and somatic cells has been reported to position the oocyte at the posterior pole in early oogenesis (Godt and Tepass, 1998; Gonzalez-Reyes and St Johnston, 1998; Riparbelli et al., 2022), the adhesive mechanisms that stabilize soma–germline contacts encountered during the massive morphological changes in late oogenesis have not been defined. In this study, we characterize the emergence and function of E-cadherin-based spot junctions between AFCs and nurse cells during late oogenesis, representing a cell biological mechanism mediating epithelial-to-non-epithelial adhesive interactions in the context of epithelial plasticity.

## 2 Results

### 2.1 Anterior follicle cells remodel adherens junctions into apical E-cadherin spot junctions with nurse cells

We first examined E-cadherin (E-cad) based adhesion of AFCs before (< stage 9) and after (> stage 9) AFCs have spread out over the nurse cell compartment (Figure 1A). We found that before stage 9, E-cadherin was organized into the typical belt-like adherens junction (AJ) structure at apicolateral surfaces, establishing a continuous adhesive network across the entire follicular epithelium (Figures 1B,B’; Supplementary Figure S1A). Strikingly, at stage 10, when AFCs have spread and established their match with the underlying nurse cells, E-cadherin was observed to have localized into spots that covered the entire interface between AFCs and nurse cells (Figures 1C,C’; Supplementary Figure S1A). Tracking the transition of E-cadherin from belt-like AJs to spots on the apical surface revealed that during stage 9, when AFCs spread over the nurse cell compartment, intraepithelial E-cadherin junctions broke down and largely vanished (Figure 1D’’), consistent with previous reports on AFC morphogenesis (Grammont, 2007). In contrast, posterior and main body follicle cells retained belt-like E-cadherin localization (Supplementary Figure S1A). We characterized E-cadherin spots in the apical AFC surface at stage 10 by staining for other AJ components, including α-catenin, β-catenin, and Bazooka, and found that these extensively co-localized with E-cadherin and with each other, defined as a nonrandom spatial overlap of the two fluorescence signals quantified using Pearson’s correlation and Manders coefficients (Figures 1E,F’; Supplementary Figures S1B, B’’). In contrast, the interface of AFCs and nurse cells was devoid of other transmembrane adhesion molecules, such as N-cadherin and echinoid (Figures 1G,G’; Supplementary Figures S1C, C’). These observations suggest that AFCs perform cell type-specific remodeling of their epithelial adhesion, whereby E-cadherin and other AJ factors are removed from lateral intra-epithelial contacts and instead relocate to the AFC’s apical interface with nurse cells.

For characterization of this interface, we performed ultrastructural analyses of stage 10 egg chambers, when AFCs had fully spread over nurse cells. We found that the nurse cell surface was rich in microvilli, filling the space between the apical surface of AFCs and nurse cells (Figure 1H). At discrete sites, however, microvilli were absent, and the membranes of both cell types extended toward each other and were directly apposed, consistent with the presence of adhesive structures (Figure 1H). Such contacts were absent in egg chambers with follicle cell- or germline-specific E-cadherin knockdown, indicating that E-cadherin was required for the formation of the observed epithelium–germline contacts (Figures 1I,J). We confirmed the organization of these contacts and the microvilli using immunofluorescence: Cell type-specific expression of *utrABD-GFP* to visualize F-actin revealed that, while AFCs presented with a finely structured F-actin network (Figure 1K; Supplementary Figures S1D, D’), nurse cells appeared with a dense F-actin meshwork, consistent with a microvillus-covered surface (Figures 1L,M; Supplementary Figures S1D–E’). This categorization as microvilli could be further confirmed by the absence of tubulin in these actin-rich nurse cell surface structures (Supplementary Figure S1F, F’’’) (Pelaseyed and Bretscher, 2018). To analyze the distribution of adhesive sites in correlation with the dense actin meshwork, we generated masks of the E-cadherin spots and subsequently randomized surrogate images. The randomized surrogate images were generated through the redistribution of the individual spot centroids randomly within the region of interest, preserving the number of spots but removing their spatial correlations (Supplementary Figures S2A–B’’’’). These images served as a Monte Carlo null model for complete spatial randomness (CSR). We found that E-cadherin sites did not correlate with dense actin sites (microvilli) and were rather localized to the edge F-actin-free regions (Figures 1M,M’, O). This is consistent with the location of broad membrane–membrane associations adjacent to microvilli-covered surfaces observed in the transmission electron microscopy (TEM) images (Figure 1H). Together, these data indicate that E-cadherin clusters represent adhesive contact sites between the apical epithelial surface and the nurse cell surface. We will refer to them as “spot junctions,” using the term descriptively, while not excluding functional analogy to spot junctions previously described (Takeichi, 2014; Wu et al., 2014; Kroeger et al., 2024).

### 2.2 Spot junction distribution is regulated and robust during substantial soma–germline interface growth

**FIGURE 2:**
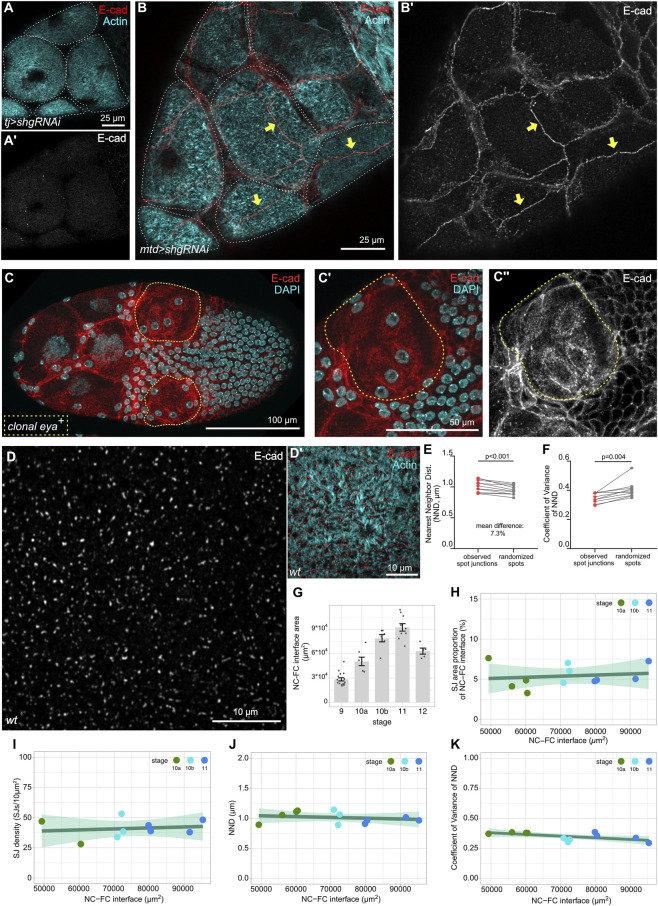
Spot junctions distribute with constant density at the follicle–nurse cell interface during growth. **(A,B)** Maximum projections of stage 10A egg chambers expressing *shg-RNAi* in follicle cells [**(A,A′)**, *TJ-GAL4*] or germline cells **(B,B’)** *MTD-GAL4*), stained for F-actin and E-cadherin. Cyan stars: AFC nuclei. Yellow arrows: AFC–AFC junctions. Note E-cadherin at AFC–AFC junctions in **(B,B’)**. **(C)** Maximum projection of an stage 10 egg chamber with follicle cell clones ectopically expressing Eyes absent (Eya, yellow dotted outline), stained for E-cadherin and DAPI (phenotype penetrance at stage 10: N = 9/10). **(C′,C’’)** show zoomed-in images of one of the clones in **(C)**. Note the lack of E-cadherin at follicle cell junctions and presence of E-cadherin spots on the apical surface. **(D,D’)** Airyscan image of the AFC–nurse cell interface in a stage 10A egg chamber stained for E-cadherin and F-actin. **(E,F)** Paired t-test of nearest-neighbor distance (NND) **(E)** and the coefficient of variance (CV) of NNDs **(F)** between E-cadherin spot junctions, observed vs. digitally randomized distributions (see Supplementary Figure S2 and Methods). Each data point represents the mean of NNDs or CV within one egg chamber, *n* = 11 egg chambers, N = 2. **(G)** Quantification of the nurse cell–follicle cell interface area at different stages. Each data point represents an egg chamber, n: stage 9 = 17, stage 10A = 7, stage 10B = 6, stage 11 = 10, stage 12 = 5, and N = 5. **(H–K)** Spot junction area proportion of the nurse cell–follicle cell interface **(H)**, spot junction density **(I)**, mean NND between spot junctions **(J)**, and coefficient of variance (CV) of NNDs within one egg chamber **(K)** as a function of the nurse cell–follicle cell interface area in *wt* egg chambers. Linear fit with a 95% CI area. n = 11 egg chambers in **(H,J,K)**; n = 9 egg chambers in **(I)** N = 2.

Expression of E-cadherin in either germline or follicle cells is required for the formation of membrane contacts between nurse cells and AFCs (Figures 1H–J). To provide additional evidence that these membrane contacts correspond to E-cadherin spot junctions, we analyzed their formation upon follicle cells (Figures 2A,A’) or germline-targeted (Figures 2B,B’) expression of E-cadherin-RNAi (*shgRNAi*). Immunofluorescence analysis confirmed the complete loss of E-cadherin spot junctions from the epithelium–germline interface in either condition. Combined, this demonstrates that the presence of E-cadherin on either surface is required for its localization of E-cadherin into epithelium–germline spot junctions, as expected from homophilic adhesion mediated by E-cadherin. To understand whether, under loss-of-function conditions, other cadherin family members might compensate for the loss of E-cadherin in epithelium–germline adhesion, we examined the localization of N-cadherin. In epithelial mosaic clones where cells are mutant for *E-cadherin*, N-cadherin enriched at intraepithelial junctions (Supplementary Figure S2C), suggesting that it compensates for the loss of intra-epithelial adhesion, as reported before (Grammont, 2007). Yet, N-cadherin did not localize to the epithelium–germline interface (Supplementary Figures S2C’), consistent with the absence of N-cadherin expression in the germline and, thus, the lack of competence for homophilic N-cadherin adhesion (Loyer et al., 2015).

Interestingly, germline knockdown not only prevented spot junction formation but also led to relocalization of E-cadherin to intraepithelial junctions of AFCs, suggesting that the driving force of spot junction formation is the ability of nurse cells to sequester E-cadherin presented by AFCs into spot junctions via homophilic binding to nurse cell E-cadherin (Figures 2B,B’). The spreading of AFCs substantially increases their contact interface with nurse cells while concomitantly reducing the lateral intra-epithelial surface. We, therefore, investigated whether the apical localization and stabilization of E-cadherin in AFCs arise from an area-dependent probability of homophilic interactions. To test this, we increased the apical surface of MBFCs, which normally reposition onto the oocyte and do not form spot junctions with nurse cells. By ectopically expressing Eyes absent (Eya) in MBFC clones, we induced ectopic cell spreading of MBFCs, which led to an increased apical surface area and reduced lateral surfaces (Weichselberger et al., 2022). We found that this was sufficient to induce E-cadherin clusters between the apical surface of MBFCs and nurse cells (Figures 2C–C’’). While we cannot distinguish if Eya-dependent AFC cell fate or the surface area increase *per se* promotes apical E-cadherin clustering and spot junction formation, we emphasize that it is the contact with nurse cells that drives Eya-dependent apical spreading; in contrast, Eya-expressing clones located over the oocyte do not increase their apical surface and do not present with apical E-cadherin clusters (Supplementary Figures S2D–E’’) (Weichselberger et al., 2022).

To better characterize E-cadherin spot junctions between these two cell types, we quantified the distribution of E-cadherin spot junctions over the epithelium–germline interface. To this end, we created a mask of the E-cadherin signal and measured the average nearest neighbor distance (NND), which was approximately 1 µm. To test whether spot junctions were randomly distributed, we compared the NND of the observed spot junctions with the NND measured within the randomized surrogate images (Supplementary Figures S2A, B’’’’). We found that the randomized NND was significantly smaller than the experimentally observed one (Figures 2D,E). Furthermore, the coefficient of variation (CV), which quantifies the relative spread of NNDs by normalizing their standard deviation to the mean, was smaller in the observed spot junctions than in the randomized surrogate images. (Figure 2F). This finding suggests a robust regulatory mechanism that ensures uniform spot junction distribution during stage 10. In addition, the localization of E-cadherin spot junctions specifically to the edge of microvilli-rich regions (Figures 1N,O, 2D’) suggests that E-cadherin spot junctions and microvilli may impact each other’s distribution during this stage.

Importantly, the interface between AFCs and nurse cells grows not only through the expansion of AFCs over the nurse cells but also through a dramatic increase in the entire egg chamber volume during stages 10a to stage 11 (Figure 2G). Remarkably, the organization of E-cadherin spot junctions was not affected by the substantial increase in the surface and, thus, interface area. Spot junction density, coverage, and spacing remained constant, as well as the variance of NNDs (Figures 2E–H,H–K). Thus, the spatial distribution of E-cadherin spot junctions appears to be tightly regulated to retain a robust distribution over the shared interface despite a dramatic increase in the surface area.

### 2.3 E-cadherin spot junctions facilitate AFC–nurse cell adhesion during nurse cell dumping

**FIGURE 3:**
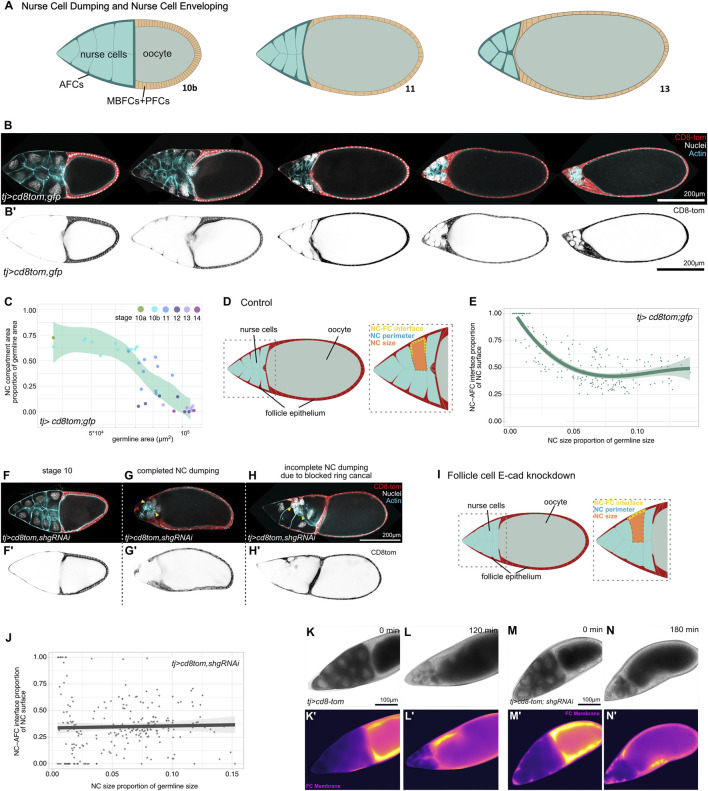
Spot junctions are required for AFC envelopment of nurse cells. **(A)** Schematic of egg chambers from stages 10B–13 during nurse cell dumping. AFCs (dark green) fully envelop nurse cells (cyan). **(B,B’)** Medial confocal sections of egg chambers during dumping, expressing *CD8::Tomato* and GFP in follicle cells (*TJ-GAL4*), stained for DNA (DAPI) and F-actin. **(C)** Quantification of nurse cell dumping in control egg chambers. Nurse cell cluster size plotted relative to total germline size. LOESS fitted with a 95% CI area. *n* = 35 egg chambers (*tj > cd8tom;gfp*), N = 3. **(D)** Schematic of the quantification method for the nurse cell–AFC interface. Orange: nurse cell area; yellow dotted line: NC–AFC interface, green dotted line: NC perimeter. **(E)** Quantification of the nurse cell–AFC interface proportion of individual nurse cells as a function of nurse cell size (proportion of NC area of germline area) in control egg chambers expressing *CD8::Tomato* and *GFP* under the control of *TJ-GAL4* in follicle cells. Cubic polynomial regression with a 95% CI area. n = 209 nurse cells from 30 egg chambers, N = 3. **(F–H)** Medial confocal sections of egg chambers during dumping, expressing *CD8::Tomato* and *shg-RNAi* in follicle cells, stained for DNA (DAPI) and F-actin. A stage 10 egg chamber **(F,F’)** and two nurse cell dumping phenotypes are shown, without **(G,G’)** and with blocked ring canals **(H,H’)**. Yellow arrowheads point to examples of nurse cells completely detached from follicle cells. **(I)** Schematic of egg chambers expressing *shg-RNAi* in follicle cells during dumping. Orange: nurse cell area; yellow dotted line: NC–AFC interface, green dotted line: NC perimeter. **(J)** Quantification of the nurse cell–AFC interface proportion of individual nurse cells as a function of nurse cell size (proportion of the NC area of the germline area) in egg chambers expressing *shg-RNAi* in follicle cells. Linear regression with a 95% CI area. n = 244 nurse cells from 37 egg chambers, N = 4. **(K–N)** Live imaging stills of egg chambers expressing *CD8::Tomato* and *GFP* **(K–L’)** or *CD8::Tomato* and *shg-RNAi (E-cadherin RNAi)* **(M–N’)** in follicle cells during nurse cell dumping. Brightfield and *CD8::Tomato* signals are shown. Note how follicle cells (FC membrane equals *CD8-Tom signal*) envelop nurse cells in **(L’)** and fail in **(N’)**.

We investigated the function of adhesive sites at the epithelium–germline interphase. The role of AFCs is to envelop each nurse cell after nurse cells have transferred their cytoplasmic contents into the oocyte and induce phagoptosis to clear the egg chamber of all nurse cell remnants (Figures 3A–C). This is an essential process of oogenesis as it ensures the production of a single germ cell, the oocyte, for fertilization. We quantified this process by measuring the proportion of each nurse cell’s surface covered by AFCs in relation to the size of nurse cells (Figure 3D). We found that the increasing shrinkage of nurse cells strongly correlated with an increasing envelopment of nurse cells by AFCs (Figure 3E).

We hypothesized that E-cadherin spot junctions may facilitate the enveloping and engulfment of nurse cells by AFCs. In egg chambers with follicle cell-specific E-cadherin knockdown, dumping was initiated normally but failed to complete (Supplementary Figure S3A). Quantification of wild-type egg chambers showed that the nurse cell area proportion of the germline decreased from ∼65% to 0%, whereas in knockdowns, it plateaued at ∼33% (Supplementary Figure S3A, Supplementary Movie S1). We found that the arrest of nurse cell dumping was due to blocked ring canals at the nurse cell–oocyte interface (Supplementary Figures S3B-D, Supplementary Movie S2), likely due to impaired centripetal cell migration between nurse cells and oocyte, a phenomenon shown to be E-cadherin-dependent (Oda et al., 1997; Hackney et al., 2007; Logan et al., 2022; Parsons et al., 2023). Blocked ring canals impair the flow of cytoplasmic contents between germline cells and the oocyte and lead to an increased variance of nurse cell sizes, probably related to nurse cell Laplace dynamics (Figures 3F–H) (Imran Alsous et al., 2021). In our study experiments, ∼90% of E-cadherin RNAi-expressing egg chambers displayed such blocked ring canals (Supplementary Figure S3B). Blockage was scored by manually inspecting z-stacks of individual egg chambers and identifying cases in which a nurse cell nucleus occluded a ring canal. Yet, importantly, in the remaining egg chambers without blocked ring canals, we observed coordinated nurse cell dumping, which suggested that E-cadherin spot junctions *per se* are not essential to facilitate cytoplasmic content transfer from nurse cells to the oocyte (Figures 3F–G’). However, we found that the envelopment of nurse cells by AFCs was strongly disrupted (compare Figure 3G’ with Figure 3B’). Specifically, in controls, the contact proportion to AFCs never decreased below 25% of the nurse cell surface and increased as nurse cells shrank, culminating in complete envelopment (Figures 3D,E). In contrast, nurse cells without blocked ring canals but lacking E-cadherin spot junctions failed to increase envelopment during dumping (Figures 3I,J). Many nurse cells displayed contact areas below 25%, and several nurse cells even became completely detached from AFCs (Figures 3G–J). Live imaging of egg chambers with labeled follicle cells during dumping further corroborated these results (Figures 3K–N’). We, therefore, concluded that spot junctions between AFCs and nurse cells are essential to maintain AFC–nurse cell contact and facilitate the complete envelopment of NCs by AFCs.

## 3 Discussion

Taken together, our results reveal a striking remodeling of epithelial characteristics in AFCs during late oogenesis. As AFCs spread over the nurse cell compartment, they dismantle intraepithelial AJs and reorganize E-cadherin into apical spot junctions at the epithelium–germline interface. Through this transformation, AFCs repurpose their apical domain into a specialized adhesive surface. This form of functional epithelial plasticity facilitates soma–germline coordination during the rapid morphological transitions in late oogenesis and provides a model for how epithelia establish adhesion junctions to stabilize interactions with non-epithelial cell types. Our work further demonstrates how adhesive plasticity allows epithelial cells to respond to their environment and fulfil different functions throughout development.

Previous work has revealed extensive roles for E-cadherin during egg chamber development: within the follicle epithelium, E-cadherin adhesion fulfills expected roles in maintaining epithelial and junctional integrity (Oda et al., 1997; Balaji et al., 2019). Between nurse cells, E-cadherin stabilizes interdigitating microvilli surrounding the ring canal opening, thereby supporting their positioning (Oda et al., 1997; Loyer et al., 2015). Homophilic E-cadherin adhesion between germline cells and follicle cells ensures the correct positioning of the oocyte during early oogenesis (Godt and Tepass, 1998; Gonzalez-Reyes and St Johnston, 1998; Riparbelli et al., 2022), as well as correct border cell migration (Cai et al., 2014) and centripetal cell ingression (Parsons et al., 2023) during later stages. While border cells and centripetal cells undergo epithelial–mesenchymal transition to become migratory, AFCs instead repurpose their apical domain to form stable contacts with a non-epithelial lineage. Our work thereby provides a novel example of epithelial plasticity in which stable adhesive coupling is redistributed from intra-epithelial junctions to an inter-lineage interface during morphogenesis.

E-cadherin clustering into the observed spot junctions could arise from a combination of cell biological mechanisms. For example, the AFC surface area increase during stage 9 likely drives the disruption of AFC intra-epithelial contacts (Grammont, 2007), thereby mobilizing E-cadherin into trafficking and sequestration at other cellular contacts. Strikingly, upon knockdown of E-cadherin in nurse cells, it remains in intra-epithelial AFC contacts, demonstrating that a major driving force of spot junction formation is the ability of nurse cells to access E-cadherin on the apical AFC surface and sequester it into spot junctions via homophilic binding, consistent with studies on E-cadherin stabilization in functional adhesion junctions (Brasch et al., 2012; Takeichi, 2014). We suggest that a combination of disrupting intra-epithelial AFC junctions, endocytic trafficking and recycling into an increasing apical AFC surface, and sequestration by nurse cell E-cadherin into spot junctions underlies the dynamics of epithelium–germline spot junction formation.

The apical localization of E-cadherin-based spot junctions in follicle cells represents an intriguing deviation from classical epithelial architecture, where apical surfaces typically face luminal spaces. From invertebrates to vertebrates, this setup can be found during oogenesis, suggesting the need for specialized adhesion mechanisms to accommodate the unique demands of oogenesis. Our findings add to the growing body of evidence that epithelial plasticity and apical–basal polarity diversification are central to the functional specialization of follicle cells in *Drosophila* and other species (Eckelbarger and Hodgson, 2021; Spradling et al., 2022). These findings have broader implications for understanding adhesion-mediated communication and coordination in other developmental systems.

## 4 Materials and methods

### 4.1 *Drosophila* stocks and genetics

All experiments were performed on *Drosophila melanogaster*. Stocks (Supplementary Table S1) and experimental crosses were maintained on standard fly food (10 L water, 74.5 g agar, 243 g dry yeast, 580 g corn meal, 552 mL molasses, 20.7 g Nipagin, and 35 mL propionic acid) at 18 °C, 22 °C, and 25 °C. Adult female *Drosophila* were fed additional dry yeast and were dissected 48–72 h after eclosion. Mosaic analysis was performed using the “flip-out” and the mitotic FLP/FRT system (del Valle Rodriguez et al., 2011). To generate mosaic follicles carrying clones of *shg* ^*R96*^, we crossed *hsflp [122]; FRT42D* ubi-nls-GFP female to *FRT42D shgR96* male *Drosophila*. Progeny of these crosses were heat-shocked at pupal stages for 1 h at 37 °C to induce recombination. To generate flip-out clones, male *Drosophila* carrying UAS-eya were crossed to *hsflp [122], tub > CD2>Gal4 UAS-GFP* female flies. Adult progeny were heat-shocked for 4–20 min at 37 °C, 48 h after eclosion, and ovaries were dissected 24 h later.

### 4.2 Immunohistochemistry and imaging

Ovaries were dissected and fixed in 4% paraformaldehyde/PBS for 15 min at 22 °C. Washes were performed in PBS +0.1% Triton X-100 (PBT). Samples were incubated with primary antibodies in PBT overnight at 4 °C: mouse anti-β-catenin (1:100, DSHB, N27A1), rat anti-E-cadherin (1:50, DSHB, DCAD2), rabbit anti-GFP (1:200, Thermo Fisher, G10362), rat anti N-Cad (1:20, DSHB, DN-Ex #8), and mouse anti-PKC ζ (1:50, Santa Cruz, H-1,sc-17781). Ovaries were incubated with secondary antibodies for 2 h at 22 °C. DAPI (0.25 ng/μL, Sigma) and phalloidin (Alexa Fluor 488, Alexa Fluor 647 and Alexa Fluor 555, Molecular Probes, or Phalloidin-TRITC, Sigma) were used to visualize DNA and filamentous actin. The following secondary antibodies were used: goat anti-mouse Alexa Fluor 488 (Abcam, AB150117, 1:500), goat anti-rat Alexa Fluor 488 (Abcam, AB150153, 1:500), goat anti-rabbit Alexa Fluor 488 (Invitrogen, A11008, 1:500), donkey anti-mouse Alexa Fluor 555 (Abcam, AB150110, 1:500), donkey anti-rat Alexa Fluor 555 (Abcam, AB150154, 1:500), donkey anti-mouse Alexa Fluor 647 (Abcam, AB150111, 1:500), donkey anti-rat Alexa Fluor 647 (Abcam, AB150155, 1:500), and goat anti-rabbit Alexa Fluor 647 (Invitrogen, A21244, 1:500). Samples were mounted using Molecular Probes Antifade Reagents.

### 4.3 Live imaging of nurse cell dumping

Ovaries were dissected into Schneiders Medium with FBS and insulin (7.6 mL Schneiders Medium, 2 mL FBS, and 0.4 mg/mL insulin, at pH = 6.9–7). Ovarioles were gently pulled out of the muscle sheet by pulling on the germarium and transferred into an imaging dish (MatTek dish) with the same medium. Stage 10b egg chambers were selected for imaging based on their morphology (ovary of similar size to the nurse cell compartment). Egg chambers were imaged on an Axio Observer 7. The time of the videos corresponds to the visual onset of nurse cell dumping.

### 4.4 Image acquisition, analysis, and quantification

Images were obtained with a LEICA TCS SP8 using software LAS X for confocal imaging, ZEISS LSM 880 Examiner for Airyscan imaging, and Axio Observer 7 for live-cell imaging of nurse cell dumping. Images were processed in FIJI (Schindelin et al., 2012). Statistical analysis and generations of graphs were performed in R (R version 4.0.5) and GraphPad (GraphPad Prism 9). All data points used for quantification are referenced in Supplementary Table S2.

### 4.5 Co-localization analysis

Co-localization of fluorescence signals was analyzed in Fiji/ImageJ using the Coloc2 plugin. A maximum-intensity projection of 2-3 sections was obtained for each image and processes by background subtraction. For each AFC, analysis was restricted to a square 50 µm x 50 µm region of interest (ROI) outside the AFC nucleus. Automatic Costes thresholding and Costes randomization were applied as implemented in Coloc2. Co-localization was quantified using Pearson’s correlation coefficient (Pearson’s r) as the primary measure of pixel-intensity correlation, together with Manders’ coefficients (M1 and M2) as measures of signal overlap. Co-localization coefficients were calculated for each AFC and pooled as biological replicates for statistical analysis.

### 4.6 Spot junction distribution quantification

The right plane of Airyscan images of spot junctions was selected, with up to three planes as Max. Proj. E-cadherin and b-cat channels were merged to create a spot junction channel. The measurement area was generated manually to exclude out-of-focus regions, and the spot junction channel was thresholded using the triangle or moments method. The despeckle function was applied. The Analyze particles function with 0.05 µm^2^–Infinity was applied. Centroid and area measurements were performed. ROIs were added to the ROI manager. NND was measured. All ROIs were combined into single ROI using the combine function and then saved as spot junctions ROI. A mask was generated from the spot junction ROI and saved as the spot junction mask. 3D Object Counter without numbering was applied. The output was directly used for 3D shuffle. The original spot junction mask was shuffled five times with the applied measurement area and saved each time (Supplementary Figure S2). Shuffled images were binarized. Then, the same procedure as for the original image was performed to extract coordinates, NNDs, and sizes.

### 4.7 Germline size, nurse cell size, and nurse cell–follicle cell interface area quantification

Egg chamber morphology measurements were performed in FIJI, using the polygon and line tools. The germline area was determined in 2D medial cross-sections (through the anterior and posterior pole (Weichselberger et al., 2022). The nurse cell–follicle cell (NC-FC) interface was calculated by approximating the nurse cell compartment as a cone and calculating the lateral surface area of a cone using the measured radius and slant height from a 2D medial section of the egg chamber. Individual nurse cell sizes were quantified as areas in confocal sections through the center of the nurse cell. Wild-type and control egg chambers were staged according to previously described criteria (Koch, 1963; Horne-Badovinac and Bilder, 2005).

### 4.8 Electron microscopy

Isolated ovarioles were incubated in Schneider’s medium containing 2.5% glutaraldehyde and 2% paraformaldehyde for 30 min, followed by incubation in PHEM buffer with fixatives for additional 30 min. Samples were flat embedded in 2% low melting agarose and placed at 4 ° C for 10 min until polymerization. Blocks of agarose were transferred to PHEM buffer with fixatives at 4 °C overnight. Post-fixation, embedding, and imaging were performed as described in Schuessele et al. (2016).

## Data Availability

The original contributions presented in the study are included in the article/Supplementary Material; further inquiries can be directed to the corresponding authors.
