# Supplementary figures and images for: Reorganization of E-cadherin into apical spot junctions mediates interlineage adhesion between epithelial and germline cells
Source: Front Cell Dev Biol. 2026 May 11;14:1807574. doi: 10.3389/fcell.2026.1807574 (PMC13199287; doi:10.3389/fcell.2026.1807574)

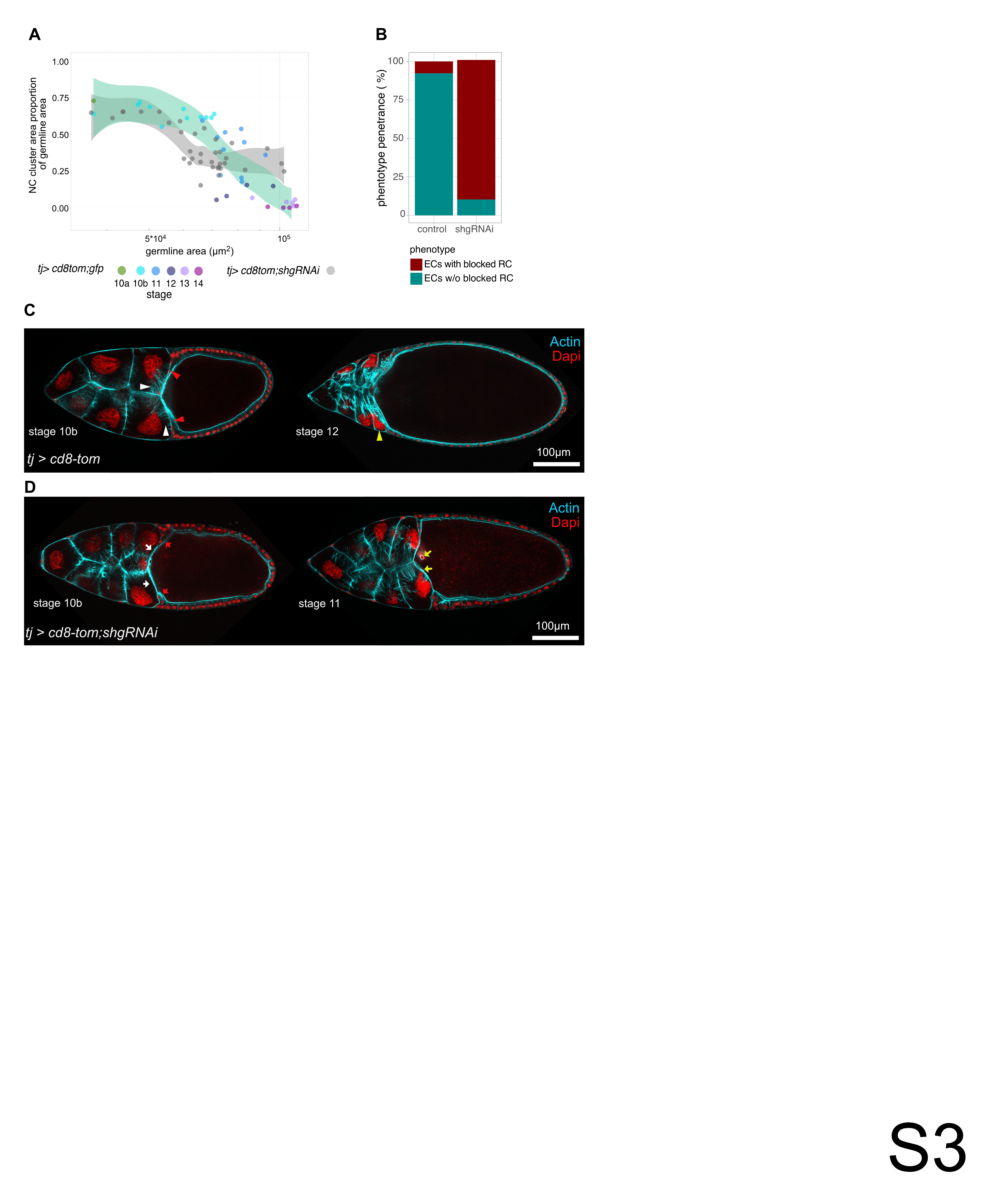

Supplement: Supplementary file 1 [file Image3.TIFF]

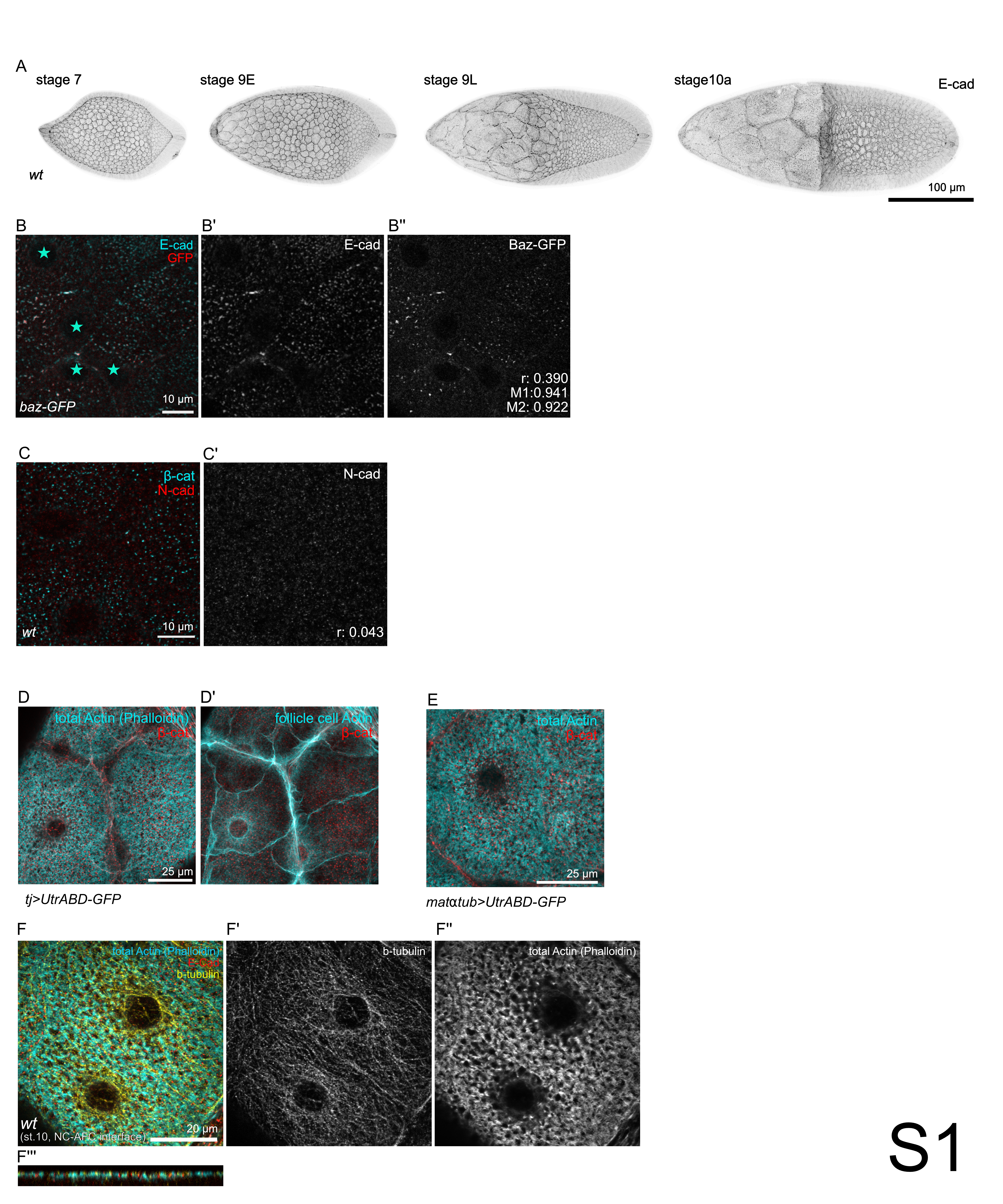

Supplement: Supplementary file 4 [file Image1.TIFF]

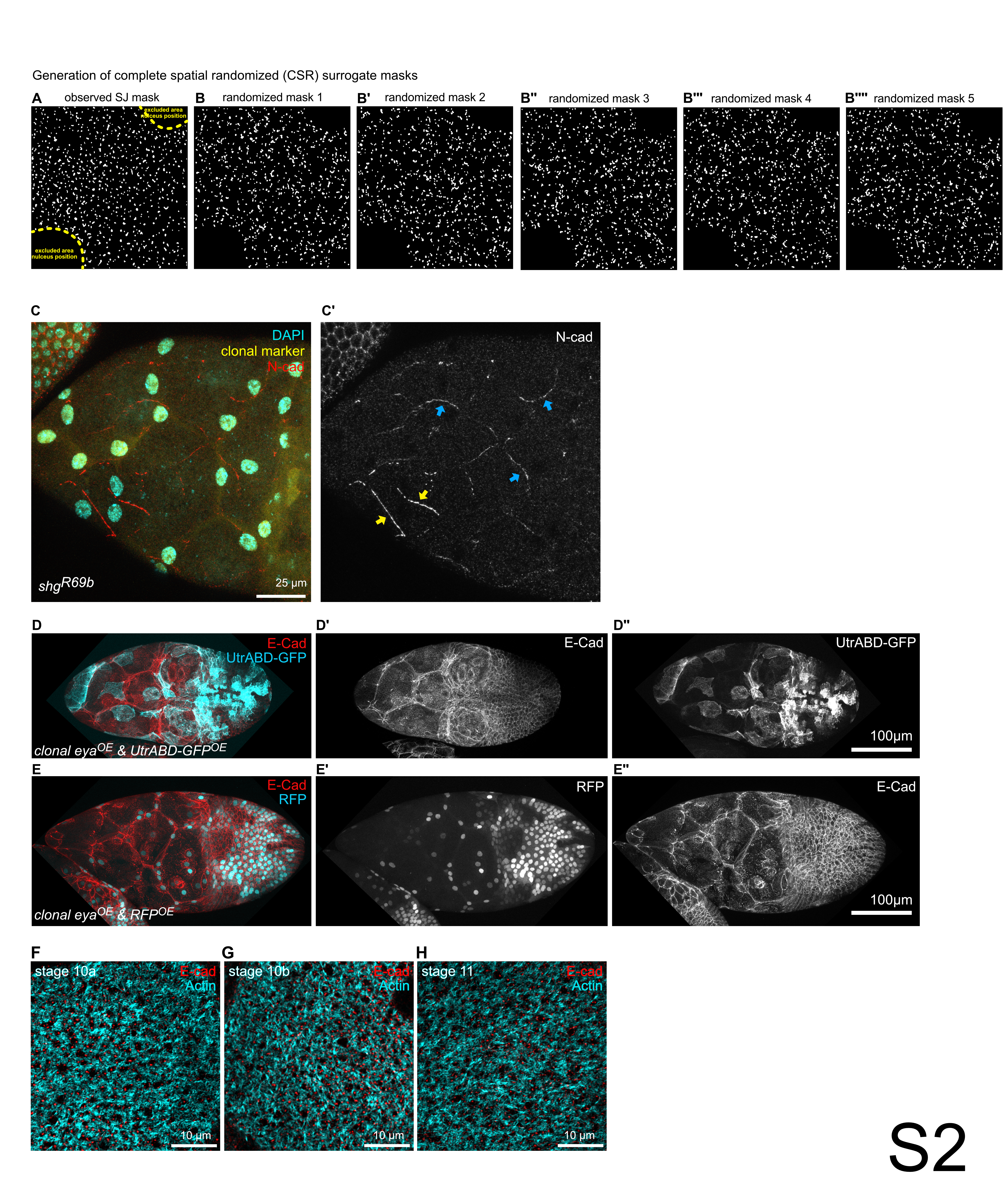

Supplement: Supplementary file 8 [file Image2.TIFF]
