# Supplementary material for: Reorganization of E-cadherin into apical spot junctions mediates interlineage adhesion between epithelial and germline cells
Source: Front Cell Dev Biol. 2026 May 11;14:1807574. doi: 10.3389/fcell.2026.1807574 (PMC13199287; doi:10.3389/fcell.2026.1807574)
Supplement: Supplementary file 5 [file Table1.pdf]

Supplementary Table S1 - Fly strains

**Fly line**	**Chromosome**	**Source**
w[118]	*X*	David Bilder
hsflp[122]	*I*	Iswar Hariharan
act>y[+]>-GAL4,UAS-RFP/TM6c	*III*	BDSC 30558
FRT42D ubi-eGFP/CyO	*I,II*	BDSC 5626
tj-GAL4, Mef2-GAL80/CyO	*II*	Sally Horne-Badovinac
tj-GAL4, UAS-CD8tom/CyO; UAS-dcr2/TM6C	*II,III*	David Bilder
MTD-GAL4 (Otu-Gal4::VP16;nos-GAL4;nos-GAL4::VP16)	*I,II,III*	BDSC 31777
matalpha-GAL-VP16	*III*	BDSC 7063
UASp-UtrABD-eGFP	*III*	Thomas Lecuit
UAS-eya	*III*	BDSC 5675
FRT42D shg [R69b]/ SM6b, cn#1	*II*	Ulrich Tepass
UAS-shg RNAi (HMS00693)	*III*	BDSC 32904
UAS-shg RNAi (GL00646)	*II*	BDSC 38207
UAS-GFP (S56T) /CyO	*II*	BDSC 1521
Ed-GFP	*II*	BDSC 59777

Supplementary Table S2 – Source Data

Source data for quantifications displayed in Figure 1-3. The original data points wih associated experimental IDs used to plot the graphs are included.
