# Supplementary material for: Reorganization of E-cadherin into apical spot junctions mediates interlineage adhesion between epithelial and germline cells
Source: Front Cell Dev Biol. 2026 May 11;14:1807574. doi: 10.3389/fcell.2026.1807574 (PMC13199287; doi:10.3389/fcell.2026.1807574)
Supplement: Supplementary file 6 [file Supplementaryfile1.docx]

**SUPPLEMENTS**

**Reorganization of E-cadherin into apical spot junctions mediates interlineage adhesion between epithelial and germline cells**

Vanessa Weichselberger ^*^ ^1,2,3,4,5^, Ramya Balaji ^3,4,6^, Marta Rodriguez-Franco ^4^, Anne-Kathrin Classen ^*^ ^3,4,6^

**Figure S1 Spot junction emergence**

**A** Maximum projection of confocal images of egg chambers from stages 7-10A, stained for E-cadherin. Note how MBFCs and PFCs retain classic belt-like adherens junction.

**B** Maximum projection of confocal images of AFCs in a stage 10 egg chamber, expressing *bazooka(baz)-GFP*, stained for E-cadherin and GFP. Mean Pearson’s R and Mander’s M1 and M2 for co-localisation are reported (E-cad/Baz n = 5 cells).

**C** Maximum projection of confocal images of AFCs in a stage 10 egg chamber, stained for E-cadherin and N-Cadherin. Mean Pearson’s R for co-localisation is reported (n = 9 cells).

**D,D’** Maximum projection of confocal images depicting AFCs in a stage 10 egg chamber expressing *utrABD-GFP* in follicle cells (*TJ-GAL4*), stained for total F-actin (using Phalloidin), β-catenin and GFP. **D** shows β-catenin and total F-Actin, **D’** shows β-catenin and GFP.

**E** Maximum projection of confocal images of AFCs in a stage 10 egg chamber expressing *utrABD-GFP* in the germline (*matαtub-Gal4*) stained for total F-Actin (Phalloidin) and β-catenin.

F-F’’’ Confocal section of the AFC-NC interface of a stage 10 egg chamber stained for total Actin (Phalloidin), E-Cadherin and b-tubulin. F’’’ shows xz-section through the interface.

**Figure S2 Spot junction distribution**

**A-B** Example of a segmented mask for spot junctions (**A**), generated from Airyscan confocal images, and the respective 5 digitally randomized distributions (**B-B’’’’**) used for analysis (see Methods).

**C** Maximum projection of an early stage 10A egg chamber with clones homozygous mutant for the *E-cadherin* *shg^R69b^* allele (negative for the clonal marker in yellow), stained for DNA (DAPI) and N-Cadherin (red in C, grey in C’). Yellow arrows point to intra-epithelial junctions lacking E-cadherin, blue arrows mark intra-epithelial junctions between control cells (C’). Note enrichment at mutant-mutant cell junctions (intra-epithelial compensation) but lack of N-cadherin positive punctae in the epithelium-germline interface.

**D-D’’** Overview image with clonal marker for Fig. 2C. Note that UtrABD-GFP expression was used as clonal marker. Egg chamber stained for E-Cadherin and GFP.

**E-E’’** Egg chamber with clonal expression of eya and RFP as control for Fig. 2C and Fig. S2A. clonal expression of Eya without UtrABD-GFP co-expression also leads to ectopic formation of apical E-Cadherin clusters. Egg chamber stained for E-Cadherin and with clonal marker expression of RFP.

**F-H** Maximum projection of confocal images of AFCs in stage 10A (D), stage 10B (E) and stage 11 (F) egg chambers, stained for E-cadherin and total F-Actin.

**Figure S3 Spot junctions are essential for nurse cell envelopment during dumping**

**A** Nurse cell compartment size as a function of germline size of control egg chambers expressing *CD8::Tomato and GFP* and egg chambers expressing *CD8::Tomato* and *shg-RNAi (E-cadherin RNAi)* under the control of *TJ-GAL4* in follicle cells. LOESS fitted with a 95% CI area. N: *tj>cd8tom;gfp*= 35 egg chambers, N=3, *tj>cd8tom;shgRNAi* = 37 egg chambers, N=4.

**B** Quantification of percentage of egg chambers with a blocked ring canal phenotype. n: *tj> cd8tom;gfp*= 13 egg chambers, N=3, *tj>cd8tom;shgRNAi* = 32 egg chambers, N=5.

**C**  Medial confocal section of control egg chambers expressing *CD8::Tomato* under the control of *TJ-GAL4* in follicle cell, stained for F-actin and DAPI. Red arrowheads: centripetal FCs migrating between nurse cells and oocyte. White arrowheads: actin cables blocking nuclei from ring canals. Yellow arrowhead: nurse cell nucleus pushed towards AFC–NC interface.

**D** Medial confocal section of egg chambers expressing *CD8::Tomato* and *shg-RNAi* under the control of *TJ-GAL4* in follicle cell, stained for F-Actin and DAPI. Red arrows point to centripetal follicle cells that fail to migrate between nurse cells and oocyte. White arrows point at nurse cell-oocyte interface lacking actin cables. Yellow arrows point to ring canals blocked by nurse cell nuclei.

**Movie S1 Nurse cell dumping in wild type egg chambers**

Live imaging of a wild type chamber expressing *CD8::Tomato* and *GFP* under the control of *TJ-GAL4* in follicle cells during nurse cell dumping. Brightfield (left) and *CD8::Tomato* (right) signals are shown. Note how follicle cells (*CD8-Tom*) envelop nurse cells.

**Movie S2 Nurse cell dumping in egg chambers expressing E-cadherin RNAi**

Live imaging of a wild type chamber expressing *CD8::Tomato* and *shg-RNAi (E-cadherin RNAi)*  under the control of *TJ-GAL4* in follicle cells during nurse cell dumping. Brightfield (left) and *CD8::Tomato* (right) signals are shown. Note how follicle cells (CD8-tom) fail to envelop nurse cells.
